# Structural and congenital heart disease interventions: the role of three-dimensional printing

**DOI:** 10.1007/s12471-016-0942-3

**Published:** 2017-01-12

**Authors:** L. M. Meier, M. Meineri, J. Qua Hiansen, E. M. Horlick

**Affiliations:** 1 Toronto Congenital Cardiac Centre for Adults, Toronto General Hospital, Peter Munk Cardiac Centre, University Health Network, University of Toronto, Toronto, Ontario Canada; 2 Department of Anesthesia and Pain Management, Toronto General Hospital, University Health Network, University of Toronto, Toronto, Ontario Canada

**Keywords:** Cardiology, Structural heart disease, Heart valve diseases, Congenital heart defects, Transcatheter interventions, Three-dimensional printing

## Abstract

Advances in catheter-based interventions in structural and congenital heart disease have mandated an increased demand for three-dimensional (3D) visualisation of complex cardiac anatomy. Despite progress in 3D imaging modalities, the pre- and periprocedural visualisation of spatial anatomy is relegated to two-dimensional flat screen representations. 3D printing is an evolving technology based on the concept of additive manufacturing, where computerised digital surface renders are converted into physical models. Printed models replicate complex structures in tangible forms that cardiovascular physicians and surgeons can use for education, preprocedural planning and device testing. In this review we discuss the different steps of the 3D printing process, which include image acquisition, segmentation, printing methods and materials. We also examine the expanded applications of 3D printing in the catheter-based treatment of adult patients with structural and congenital heart disease while highlighting the current limitations of this technology in terms of segmentation, model accuracy and dynamic capabilities. Furthermore, we provide information on the resources needed to establish a hospital-based 3D printing laboratory.

**Electronic supplementary material:**

The online version of this article (doi: 10.1007/s12471-016-0942-3) contains supplementary material, which is available to authorized users.

## Introduction

**Fig. 1:**
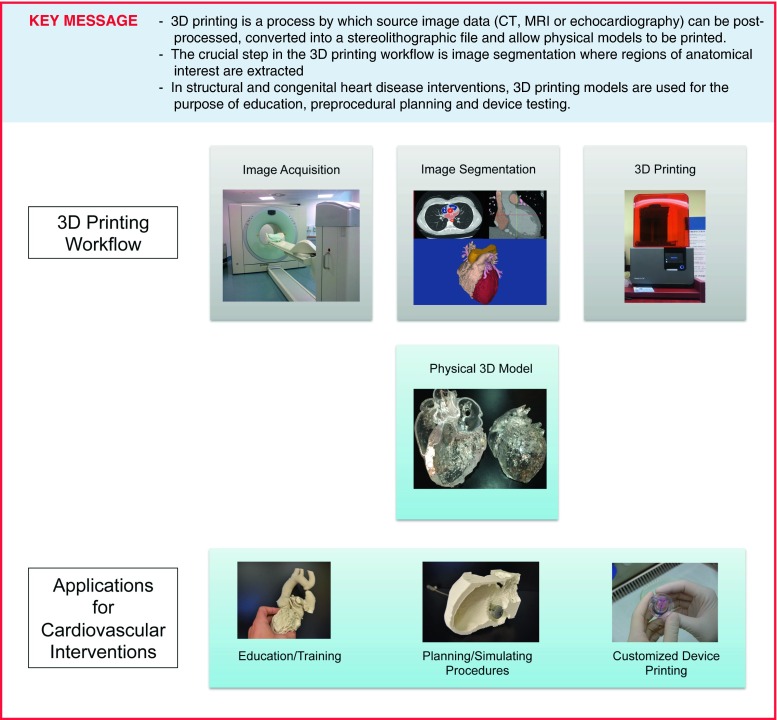
3D printing workflow and applications

Structural heart disease intervention has been a rapidly growing field in interventional cardiology, involving a broadening variety of catheter-based treatment options for acquired and congenital heart defects. Transcatheter interventions have become the standard of care for several structural and functional abnormalities of heart valves, cardiac chambers and proximal vessels [1]. Patients with congenital heart defects and complex previous operations represent a challenge due to a wide variation in morphology and complex cardiac anatomy. Despite currently available three-dimensional (3D) imaging modalities most procedures are still planned using images viewed on two-dimensional (2D) screens. The 2D representation makes it inherently more difficult to fully appreciate the complex 3D relationships of cardiac structures relevant to particular interventions. 3D printing (also referred to as rapid prototyping, stereolithography or additive manufacturing) is a technology which fabricates a physical model from a 3D computerised imaging source file. The first medical application was used to produce surgical implants for oral and maxillofacial surgery [2] and prosthetics for orthopaedic surgery [3]. The ability to generate a tangible 3D model of complex cardiac anatomy has made this a promising tool for education, preprocedural planning, and device testing in structural and congenital heart disease interventions (Fig. 1; [4–6]). However, due to the expertise required to generate 3D models and the investment of resources involved the establishment of 3D printing labs has mostly been limited to larger teaching hospitals and research centres. This review will discuss a 3D printing workflow, review the expanded applications of 3D printing in the catheter-based treatment of adults with structural and congenital heart disease and will outline the resources needed to establish a hospital-based 3D printing laboratory.

## 3D printing step-by-step

The availability of cross-sectional cardiac imaging, such as magnetic resonance imaging (MRI) or computed tomography (CT), for most patients with complex cardiovascular disease is favourable for the application of 3D reconstruction and printing. 3D echocardiography is also readily available in many clinical settings. Commonly available post-processing software allows anatomic visualisation via 3D renderings, but these renders cannot be directly printed. Special software is required to process the source imaging datasets to generate a model via segmentation and export it as a stereolithographic (STL) file to be printed (Fig. 1).

### Image acquisition

The three most commonly used medical imaging modalities for 3D image generation are CT, MRI and echocardiography. Spatial resolution, tissue contrast and slice thickness determine the quality of the 3D dataset and are a prerequisite for 3D printing. CT and MRI are the most common sources of datasets for 3D printing because of their ability to image the entire heart with detailed intracardiac anatomy. Echocardiography has a superior ability to image fast moving structures such as cardiac valves [7]. Fusion of different modalities (e. g. ventricles from CT, valves from echocardiography) to create a single 3D model has been reported [8]. Both ECG and non-ECG gated MRI have been used for 3D modelling and both seem to provide 3D printed models of comparable quality. Optimal CT images should be contrast enhanced, multiphase ECG-gated acquired during breath-hold [6]. Isotropic volumetric data should be reconstructed with a slice thickness between 0.5–1.25 mm to ease segmentation and generate accurate 3D printing models [9]. In echocardiography, conversion to a ‘Cartesian’ DICOM file is necessary for 3D model generation and this requires special proprietary software (e. g. Philips QLAB, Philips Healthcare, USA).

### Image segmentation

Segmentation is part of the post-processing sequence wherein source images are partitioned into multiple simple geometrical elements of known coordinates in 3D space. It is achieved using complex techniques such as surface triangulation, isosurfacing and volume rendering.

**Fig. 2:**
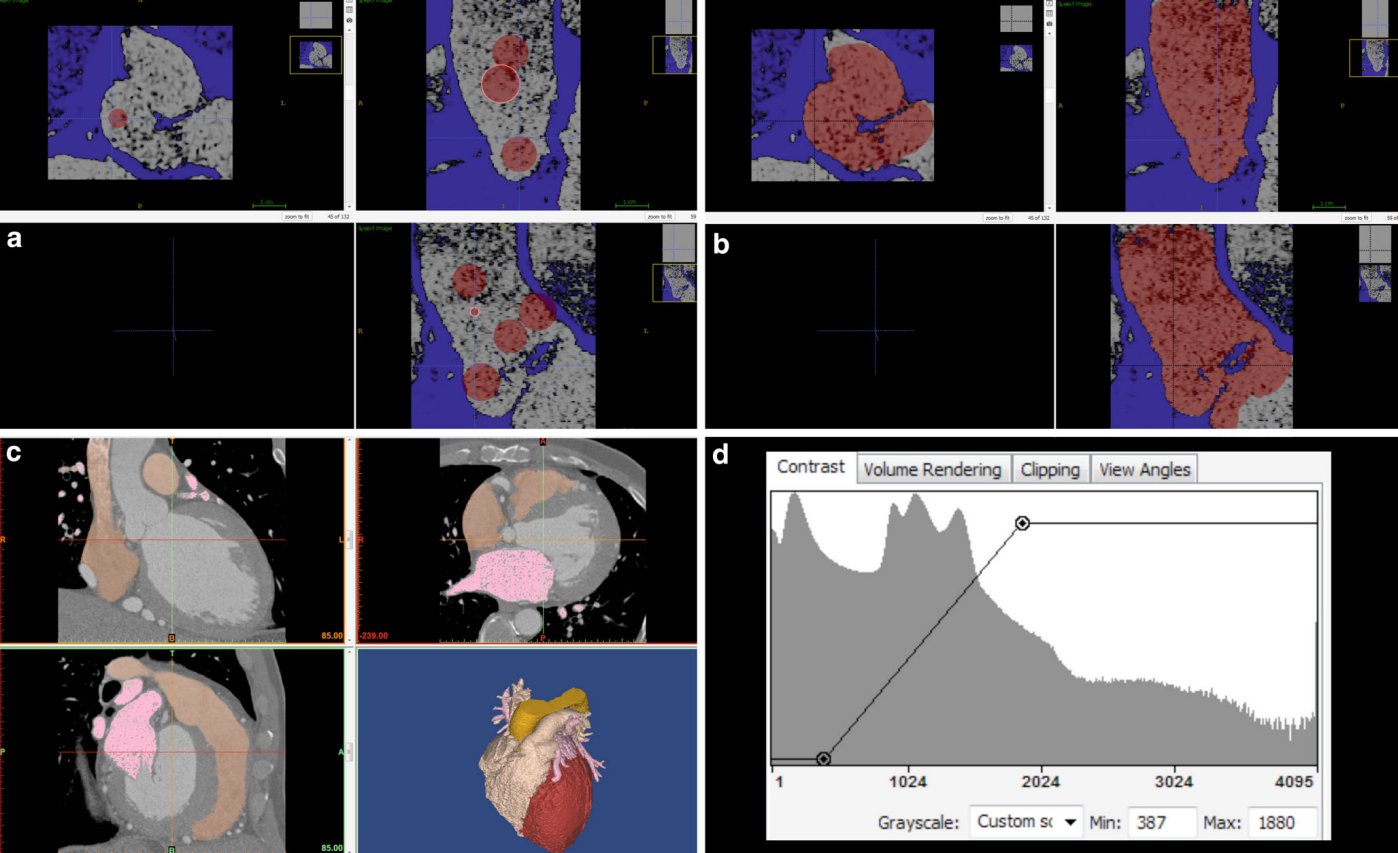
Image segmentation process. **a,b** Screenshot from ITK-SNAP-Software (http://www.itksnap.org) Region growing as part of segmentation. **a** Example of segmentation via region growth (aorta). Select the region of interest for semi-automatic active contour segmentation and laying down the red dots to define where the region of interest is. **b** Activating the algorithm causes the dots to expand into the region of interest. **c** Screenshot from Mimics-Software as an example of thresholding and manual editing. **d** The histogram window on the bottom right-hand corner is the graph where thresholding is usually set

There is no standardised approach to image segmentation. Several manual, semi-automatic and automatic image segmentation methods have been used. The most common are: region growing and brightness thresholding followed by manual editing [7]. Region growing examines the relationship of neighbouring pixels to an initial seed point and determines whether the neighbouring pixels should be added as part of that region (Fig. 2a, b). Thresholding is a method where pixels are partitioned depending on their intensity or brightness value (Fig. 2c, d). Manual editing is necessary to correct segmentations errors (exclusion of artefacts and/or filling of dropout gaps), surface smoothing, colouring and 3D model cropping. Commercially available software (Mimics, Belgium) combines semi-automatic segmentation and manual editing in a single product and allows segmentation from all imaging modalities. Freeware, such as ITK-SNAP or 3D Slicer [10, 11], is also available but is less user friendly and currently limited to CT and MRI data. The process is time consuming, requires expertise with specific segmentation software as well as understanding of the patient’s complex structural and congenital anatomy. Throughout each step, there is a risk of introducing design errors and manipulating the original source data in order to generate oversimplified 3D models [12].

### 3D printing

There are three common types of 3D printers used for medical printing. They are as defined by the mechanics and materials used to generate a model: fused deposition modelling (FDM), stereolithography (SLA) and PolyJet 3D printers. These, and other types of 3D printers, are briefly summarised in Table 1.

**Table 1:** Summary of 3D printer technologies

3D printer technology	Type of materials used	Strengths	Limitations
Fused Deposition Modelling (FDM)	Thermoplastics	Low cost, easy to operate, wide variety of usable thermoplastic materials for printing	Relatively long print times, print resolution low compared with other types of printers (0.1–1.2 mm)
Stereolithography (SLA)	Photosensitive resins	Can be low cost, high resolution (0.025–0.1 mm), excellent print surface quality	Relatively long print times, extensive post-processing required, higher end expensive industrial grade printers
Continuous Liquid Interface Production (CLIP)	Photosensitive resins	Extremely fast print speeds, high resolution prints	Printers cannot be purchased, but may be leased on a year-to-year basis
PolyJet	Photosensitive resins	Extremely high resolution (16 microns), multi-durometer printing, multi-coloured printing, large build volume	Expensive to purchase and operate, printed objects are relatively brittle
Selective Laser Sintering (SLS)	Chamber of powdered material including nylons, glass, ceramics and metal	Very large build volumes can produce mechanically functional prints out of ceramics and metals, excellent surface quality and precision	Expensive to purchase and operate, difficult to operate and calibrate

FDM printers utilise thermo-plastic filaments and a layer-by-layer technique. The filaments are rapidly heated to liquefy the plastic into a semi-molten state and then extruded in a very thin layer onto a heated surface [13]. Once extruded, the plastic quickly solidifies and fuses with the underlying adjacent layer. FDM 3D printers are capable of producing layers between 0.1 and 1.2 mm thick. A growing variety of stiff and elastomeric thermoplastics have recently become available. Desktop FDM 3D printers typically cost a few thousand US dollars and the thermoplastic filaments range from 20 to 100 US dollars per kg, making fabrication costs relatively affordable.

SLA printers utilise a photosensitive resin and ultraviolet light. The ultraviolet light traces the geometry of an object into a resin bath causing photo-polymerisation of the resin into a solid [13]. Desktop SLA 3D printers are available and priced similarly to FDM systems with a slightly higher production cost. SLA printers provide extremely high resolution ranging from 0.025 to 0.1 mm with excellent surface finish after considerable post-processing [13]. The main drawbacks of desktop SLA printers are the relatively small build volumes and long build times required to cure the photosensitive resins into 3D models. However, recent advances in printers utilising similar technology, such as Continuous Liquid Interface Production (CLIP), has dramatically reduced print times [14]*.*

PolyJet printers utilise a photo curable resin sprayed in a very fine layer and then cured into a solid via ultraviolet light [13, 14]. PolyJet printers allow extremely high resolution of 16 microns and the ability to produce regions of variable durometer within a single 3D printed object. Furthermore, objects can be produced with a wide spectrum of colour within the same print. These printers are in the order of hundreds of thousands of US dollars and have a relatively high production cost.

## Applications of 3D printing

Due to its ability to illustrate complex anatomy of cardiovascular structures and cavities, 3D printing is rapidly gaining interest in interventional cardiology and cardiovascular surgery [5]. Over the past 5 years there has been a remarkable increase in published studies and case reports for applications of 3D printing in structural and congenital heart disease interventions gap (Table 2 and 3). The applications of 3D printing vary from anatomical education and training, to preprocedural planning, simulation and device testing [15].

### Education and training

**Fig. 3:**
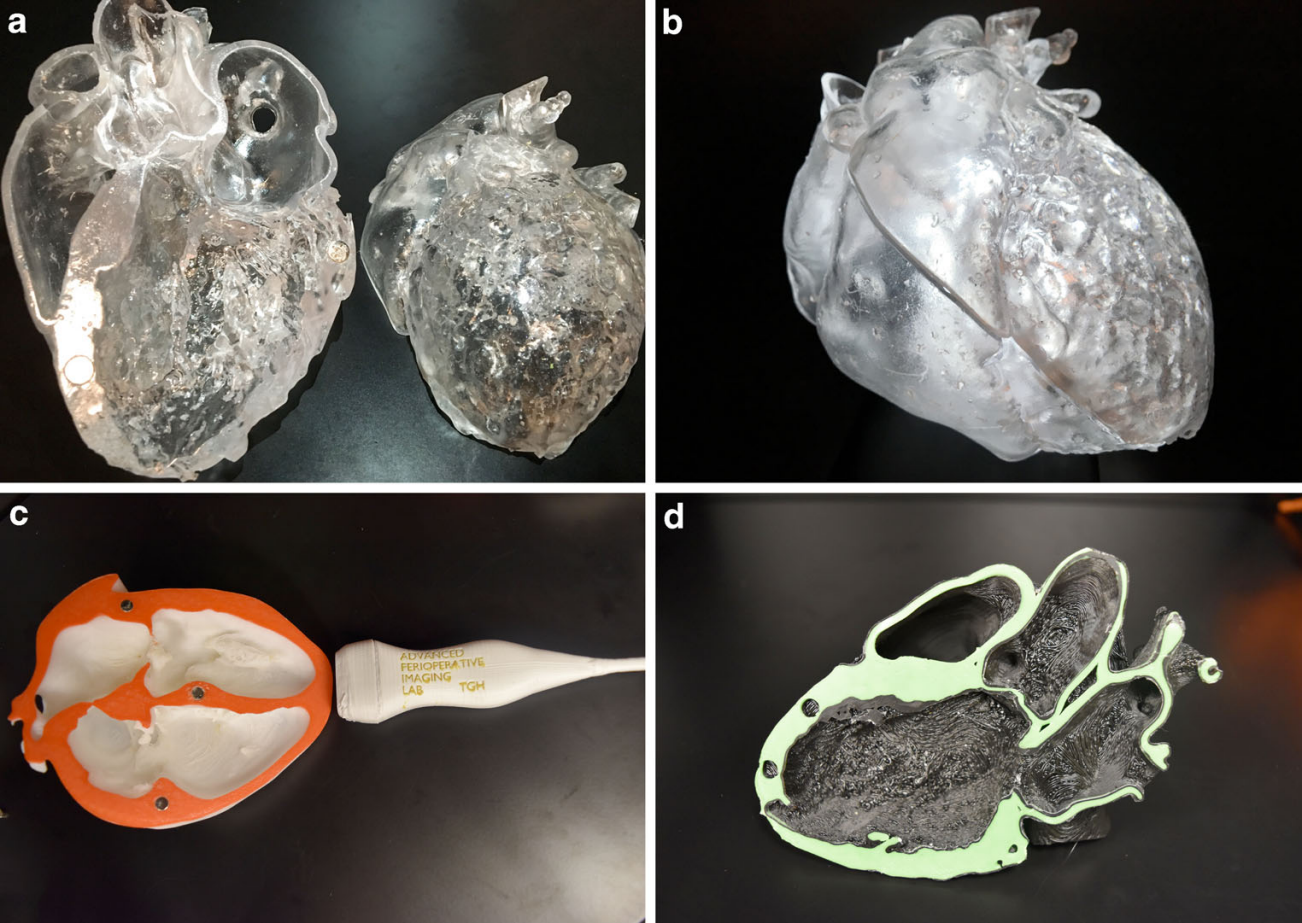
3D printing models for educational purposes. **a,b** SLA transparent full heart model demonstrating normal anatomy. **c,d** FDM models illustrating standard transthoracic echocardiographic 2D views (**c** apical four chamber view; **d** parasternal long axis)

Conceptual 3D understanding of complex cardiac structures remains a main challenge for cardiologists and radiologists and it is traditionally taught using 2D images or pathological specimens. 3D printing allows the development of accurate life-like educational tools (Fig. 3) to illustrate complex cardiovascular anatomy and pathology. In terms of qualitative assessment of knowledge reporting, knowledge acquisition and structural conceptualisation, 3D printed models have been shown to improve physicians’ understanding of congenital heart disease compared with regular imaging modalities [15]. Patient-specific 3D printing models have the potential to enhance engagement with patients and improve communication between the cardiologist and the patient’s family. Better understanding may also impact a patient’s psychological adaptation following treatment [4].

### Structural and valvular heart disease interventions

In structural heart disease interventions, the vast majority of 3D printed models have been used to assist planning and simulating complex interventions (Table 2), especially when common 2D or 3D imaging modalities raise uncertainties in terms of access relationships, or location of defects or certain structures.

**Table 2:** Overview on literature on 3D printing applications for transcatheter interventions in structural and valvular heart disease

Topic	Application of 3D printing models	Benefit
*Acquired structural heart disease*		
Coronary interventions	Coronary 3D models for percutaneous intervention (PCI) optimisation strategies [33, 34]	– In vitro simulation of PCI in complex coronary anatomy – In vitro stent placement and hydrofluid testing of haemodynamics and stent positioning – Virtual modelling of fractional flow reserve (FFR) and hyperaemic stenosis resistance index
Left atrial appendage (LAA)	3D printing models for planning/simulating LAA occlusion procedures [35, 36]	– LAA 3D models effectively guide device selection and placement of the LAA occlusion device – Optimising transseptal puncture in LAA occlusion
Great vessels	Interventions of ascending aorta: – Custom-made devices for coil embolisation of an anastomotic leak after aortic arch replacement [18] – Occlusion of an ascending aortic pseudo aneurysm [37]	– Choosing the treatment option, planning and simulating the occlusion of the pseudoaneurysm – 3D models are used to build the custom-made occluder device
	Preprocedural planning/simulating transcatheter caval valve implantation [38]	– 3D printing of the right atrial-inferior caval vein (RA-IVC) topography aids in transcatheter valve selection
*Valvular heart disease*		
Aortic valve	Preprocedural planning/simulating transcatheter aortic valve implantations (TAVI) [21, 39] – Calcium distribution patterns of the aortic valve as a risk factor for the need of permanent pacemaker implantation [40] – 3D printing of aortic roots to design tailored transcatheter stented aortic valves [41, 42]	– Patient-specific 3D models to assess the physical interplay of the aortic root and implanted valves. – 3D models may complement traditional techniques used for predicting which patients are more likely to develop paravalvular aortic regurgitation – 3D printed tissue-mimicking aortic root may enable predictions of post-TAVI root strain and distribution and aortic flow pattern
	Replicating patient-specific severe aortic valve stenosis with functional 3D modelling [22]	– Using fused dual-material 3D printing and an in vitro pulsatile flow loop demonstrates that patient-specific models can replicate both the anatomic and functional properties of severe degenerative aortic valve stenosis
Mitral valve	Preprocedural planning/simulating transcatheter mitral valve interventions [19, 23]	– Preprocedural evaluation of catheter-based repair devices within specific patient 3D printed valve geometry – Integration of CT and 3D print could assist in predicting left ventricular outflow tract obstruction – 3D models of normal and pathological mitral valve annuli before and after repair procedures
Tricuspid valve	Planning of percutaneous tricuspid interventions [43]	– 3D printing is helpful clinical tool for planning and training operators in the early stage of this innovative intervention – Measuring valvular diameters on 3D models is feasible and compared with measurements of 2D imaging and models, indicating accuracy of <1 mm
Pulmonary valve	See congenital heart disease (Table 2)

**Table 3:** Overview on literature on 3D printing applications for education and transcatheter interventions in congenital heart disease

Topic	Application of 3D printing models	Benefit
*Congenital heart disease*		
Complex congenital anatomy	Physician education and understanding of complex anatomy [15, 44]	– Effective educational tool for physicians to improve understanding of congenital cardiac anatomy – Subjective improvement of understanding of congenital heart disease with 3D printing models compared to regular 2D/3D imaging modalities (e. g. CT/MRI)
	Patient-specific 3D printing models for patient education and communication [4]	– Patient-specific models can enhance engagement with parents and improve communication between cardiologists and patient/parents
	Deriving 3D printing models from echocardiography and combined imaging modalities [8, 26, 45]	– Feasibility of deriving 3D printing from ultrasound provides an additional cost-effective and patient-centred option – Integration of the strengths of two or more imaging modalities into 3D printing is feasible and has the potential to enhance visualisation of cardiac pathomorphology
Atrial septal defect (ASD)	Preprocedural planning/simulating of transcatheter ASD closure: – secundum ASD with rim deficiency [16] – inferior vena cava type ASD with patent ductus arteriosus occlusion device [46]	– 3D printing models allowed to overcome the 3D visualisation of the ASD and guides device selection and placement
Ventricular septal defect (VSD)	– Preprocedural planning of transcatheter VSD closure for postinfarct or complex muscular VSDs [47, 48]	– Utilising 3D printing model to visualise location and size of VSD as well as trabeculations, papillary muscle bundles to guide size and type of septal occluder
Aortic coarctation	Planning/simulating endovascular stenting in transverse aortic arch hypoplasia [49]	– 3D printing models accurately replicate patients’ anatomy and are helpful in planning endovascular stenting in transverse arch hypoplasia
Transposition of the great arteries (TGA)	Hybrid 3D printing with congenitally corrected transposition of the great arteries (ccTGA) [8] Patient-specific 3D models of the cardiac chambers of a patient with D‑TGA after the Mustard operation [27]	– 3D models give important insights into the changes in size and shape of the different chambers and the patterns of blood flow from the pulmonary and systemic veins to the “appropriate” ventricle. – Helpful in understanding and optimising the overall haemodynamic function after the Mustard operation

Chaowu et al. reported a successful *in vitro* trial occlusion of an atrial septal defect (ASD) with rim deficiency on the basis of a personalised 3D printed heart model. It has shown to be a feasible method to identify the appropriate candidates, especially for large ASDs with rim deficiency, and thus decreases related complications [16]. 3D printed models have shown to be beneficial in preprocedural planning and device testing of other transcatheter interventions such as left atrial appendages occlusions [17] and interventions on the ascending aorta [18].

#### Case example (Fig. 4)

**Fig. 4:**
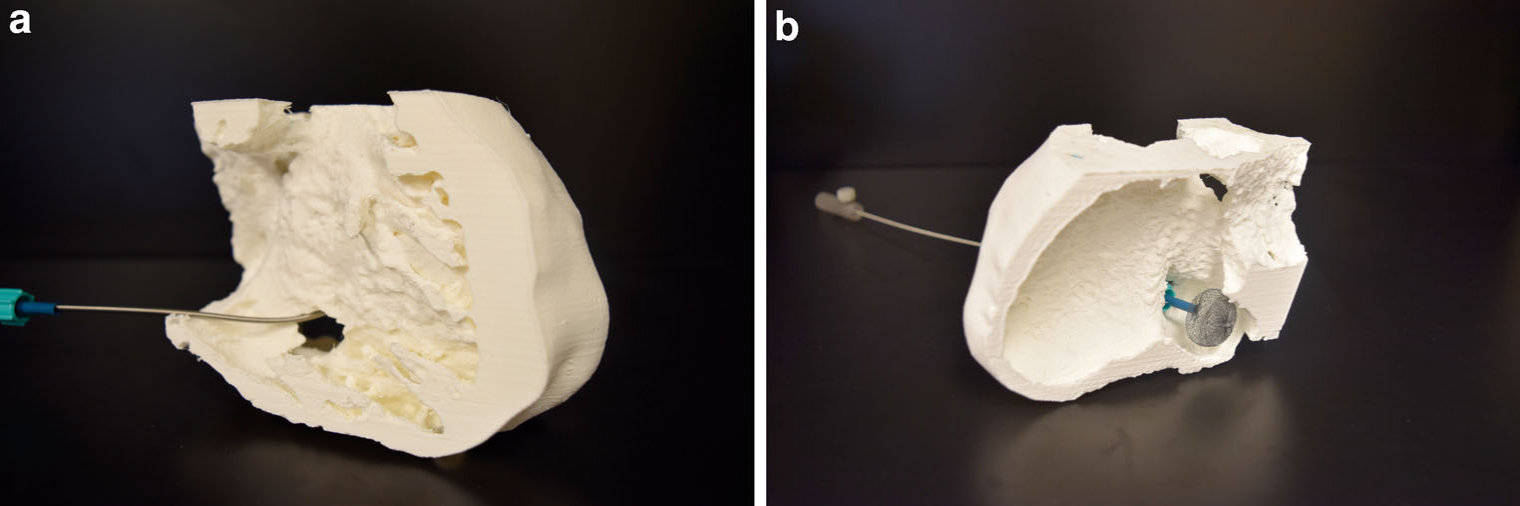
FDM/PLA – 3D printing model of an inferior post-myocardial infarction VSD with aneurysm. Amplatzer device crossing the VSD from the venous, right ventricular side. **a** right ventricular view. **b** left ventricular view

Referring to other reports, at our centre we have successfully closed a post-inferior myocardial infarction ventricular septal defect (VSD) with a post-infarct VSD occluder device based on a CT-derived 3D printed model. A 63-year-old woman with a subacute post-infarct VSD and related pulmonary hypertension and was considered for transcatheter closure. The CT revealed a large defect in the basal to mid inferior septum which measured approximately 31 × 32 × 23 mm en face. We questioned the precision of these measurements and produced a polylactic acid (PLA) FDM 3D printed model of the patient’s septum and inferior VSD. On preprocedural angiography and transoesophageal echocardiography (TEE) the maximal diameter of the VSD was between 11 and 13 mm and was consistent with the measurements and morphology of the 3D printed model. We were able to successfully deliver an 18 mm Amplatzer (St. Jude Medical Inc., USA) post-infarct VSD device (Fig. 4).

### Valvular interventions

Cardiac valves are fast moving structures that are best imaged using 3D echocardiography. One exception is severely calcified, relatively immobile aortic valves and roots that have been successfully reconstructed using CT data. Echocardiography has been used to print accurate rigid 3D models of the mitral valve and ring [19]. A flexible mitral valve has also been created on a 3D printed mould and tested in a pulse replicator [20].

Combined-material 3D printed aortic valve and root has been reported to mimic calcified regions and become an accurate tool for preprocedural testing of transcatheter aortic valve implantation and predict paravalvular regurgitation [21]. 3D printed models also seem to replicate the anatomic and functional properties of severe degenerative aortic valve stenosis in in-vitro flow systems [22]. 3D printing of cell scaffoldings is in the very early testing phase for the aortic root, and has the potential for personalised implantable device fabrication and valve tissue engineering [18, 23].

### Congenital heart disease interventions

Despite current classifications and treatment guidelines [24, 25] for adults with congenital heart disease, most of the management options are customised to individual patient anatomic variations, more so than with acquired disease. This makes patient-specific 3D printed models an ideal tool in this subset of patients for the planning of catheter-based (Table 3) and surgical interventions [4, 5, 8, 15].

Poterucha et al. reported the use of 3D right ventricular outflow tract printing and 3D rotational angiography to guide pulmonary valve stenting and valve implantation [26]. Hybrid 3D printed heart models of congenitally corrected transposition of the great arteries (TGA) [8] and dextroTGA after a Mustard operation [27] have been produced for illustrating the volume and morphologies of the chambers and proximal vessels. For those patients, 3D models could provide important insights into the changes in size and shape of the different chambers and the anatomy of vessels entering and exiting the baffles to the related ventricle.

In the future 3D printed vascular grafts for patients with congenital heart disease could be biodegradable, mechanically compatible with vascular tissues, and support neo-tissue formation and growth [28].

#### Case example (Fig. 5)

A 64-year-old patient with a history of surgical repair of supra-cardiac total anomalous pulmonary venous return with redirection of the pulmonary veins to a confluence prior to connection to the left atrium was admitted due to progressive dyspnoea on exertion. MRI and TEE showed a haemodynamically relevant stenosis between this confluence and the left atrium which led to pulmonary hypertension. Consensus was to proceed with balloon dilatation and stenting of the narrowed connection to the left atrium.

**Fig. 5:**
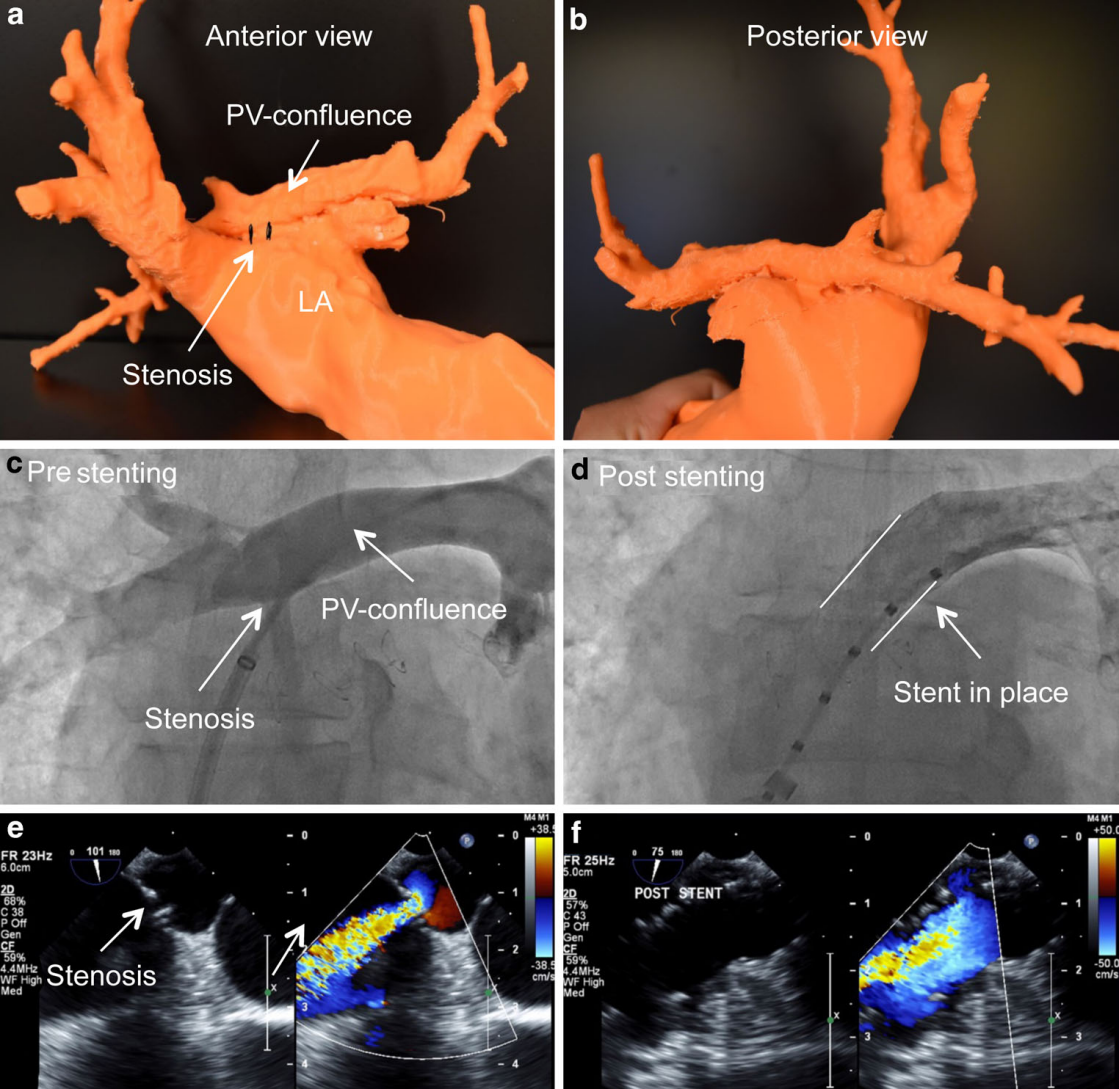
Surgical repair of a supra-cardiac total anomalous pulmonary venous connection with redirection of the pulmonary veins to a confluence prior to connection to the left atrium. Haemodynamically relevant stenosis between confluence and the left atrium. **a,b** FDM 3D printed model showing anatomy of the pulmonary venous (PV) confluence and the left atrium (LA), anterior (**a**) and posterior (**b**) view. **c,d** Periprocedural angiography. **c** Draining PV confluence and stenosis, (**d**) relief of stenosis post stenting. **e,f** Periprocedural TEE. 2D and colour flow Doppler showing relevant stenosis between PV confluence and LA with relevant proximal isovelocity surface area (PISA) before dilatation and stenting (**e**) and no relevant stenosis post stenting (**f**)

As seen in Fig. 5, the patient specific 3D printed model was especially helpful in periprocedural planning. It helped to visualise the spatial orientation of the pulmonary venous confluence, the orifice and its relation to the left atrium. After a transseptal puncture we successfully performed a balloon dilatation and a subsequent stenting of the narrowed confluence orifice (Genesis 3910B stent mounted on a 14 mm, Z‑Med II balloon) into the confluence.

## Establishing an in-hospital 3D printing laboratory

Establishing a 3D printing laboratory requires planning in three main categories: hardware, software and personnel. The necessary hardware includes: computer workstations, secured data storage, connectivity to the hospital imaging network and 3D printers. As previously described, the price of printers varies from a few thousands for desktop FDMs to a million US dollars for an industrial SLA. The following factors help guiding the choice of 3D printer: 3D print volume, turnaround time, type and need to merge of multiple materials. The costs of maintenance, printing materials and development cycle of new technologies have to be considered in this planning stage. Low cost desktop printers have significantly improved their performance over the past few years and allow affordable prototyping, making them ideal throughout the learning phase of segmentation and printing. Outsourcing single, more complex, prints should be considered as well as acquiring printers in stages as the applications and workload demand.

A range of free, open-source software packages can be part of the processes of segmentation, model editing, *de novo* modelling and slicing for 3D printing. These include software which runs effectively on modern laptops and desktops in Windows and Mac OS environments. 3D Slicer [11] and ITK-SNAP [10, 29] are typically used for segmentation and Meshmixer (Autodesk Inc., USA) for model editing. Slicing for 3D printing can be performed with a variety of software such as Slic3r (GNU Affero General Public License, version 3) or PreForm software (Formlabs, USA) depending on the printer being used. *De novo* modelling and computer-aided design work can be performed with tools such as Blender [30] and FreeCAD [31]. Mimics segmentation software (Materialise, Belgium) allows semi-automated segmentation and 3D modelling on echocardiographic datasets. Mimics is proprietary and has a yearly licensing fee.

Lab staffing depends upon the workload, the complexity and type of the models generated and the imaging modality used. As an example, CT-based 3D model of bony structures are far less time consuming than echo-based models. A biomedical industrial designer who is trained in performing 3D segmentations would be the ideal key player in the lab but will need to work in direct contact with an imaging or clinical medical specialist to guarantee the accuracy of the end products.

## Limitations and challenges

Several limitations exist with current 3D printing technologies. First, 3D printed objects are limited in replicating the dynamic properties of the organs which they seek to replicate [32]. While PolyJet printing technology can produce objects with regions of varying properties, full replication of the mechanical properties of cardiac tissue and its complex, anisotropic structure has yet to be achieved. Secondly, current image segmentation software requires a great deal of non-automated human input to produce a 3D model capable of being printed. This has a major impact on resource utilisation, as it requires dedicated trained personnel who are difficult to justify at the moment for current experimental clinical application. Additionally, this process is time consuming and is prone to human operator error and bias. This raises the issue of consistency in model accuracy. Similarly, the time required to print even a small isolated portion of the heart is in the order of at least one hour with most available printers which limits the viability of 3D printing for urgent cases. Therefore cost-effectiveness, model accuracy and clinical impact require more research and scientific data.

Lastly, visualising complex intracardiac lesions or guiding devices while simulating procedures sometimes requires the printed models to be pellucid or have static cut planes depending on the viewing angle. These can lead to time consuming reproduction of different 3D printing models for the same lesion and thus getting away from its original purpose of a comprehensive model.

Emerging virtual reality 3D imaging technologies such as Echopixel (EchoPixel, Inc., USA) or Real View Medical Holography (RealView Imaging Ltd., Israel) could be complementary tools to address those limitations. Whereas the segmentation process of the acquired images mainly remains the same for 3D printing and 3D virtual imaging, the STL files of the virtual 3D models can easily be uploaded into these virtual reality 3D tools to optimise illustration and the viewing angle of lesions by adding cutting planes. However, the tangible aspect and mimicking of tissue behaviour can only be assessed with a physical model.

## Conclusion

3D printing is a very powerful tool in structural and congenital heart disease interventions. It overcomes some of the limitations of conventional 2D/3D imaging methods by providing a tangible, physical 3D model of complex cardiovascular structures. 3D models have shown promising results in widespread applications from education to procedural planning and device testing. However, most of the current literature is based on feasibility studies and subjective qualitative comparisons with traditional imaging modalities. The role of 3D printing models in day-to-day clinical care as it pertains to morbidity and mortality is yet to be determined. More work is finally needed to automate and standardise the image segmentation process to decrease costs of personnel and allow reproducible findings. Lastly, evolving visualisation software solutions that can generate holographic 3D models, therefore acting as “virtual” 3D prints, are highly complementary to 3D printing especially because they allow dynamic rendering and cutting planes to optimise viewing angles.

## Caption Electronic Supplementary Material

Comprehensive literature search results
