# Supplementary material for: Structural and congenital heart disease interventions: the role of three-dimensional printing
Source: Neth Heart J. 2017 Jan 12;25(2):65–75. doi: 10.1007/s12471-016-0942-3 (PMC5260628; doi:10.1007/s12471-016-0942-3)
Supplement: Supplementary file 1 — Comprehensive literature search results [file 12471_2016_942_MOESM1_ESM.docx]

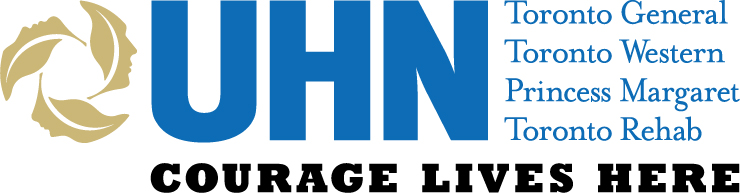


# UHN Health Sciences Libraries

# Literature Search Results

| \| **Ovid MEDLINE(R)**1946 to July Week 1 2016 \| \| \| \| \| --- \| --- \| --- \| --- \| \| **#** \| **Searches** \| **Results** \| **Type** \| \|  \| \| \| \| \| \| \| \| 1 \| Printing, Three-Dimensional/ \| 627 \| Advanced \|  \|  \|  \| \| 2 \| (three dimension* adj3 (print* or model* or manufactur*)).ti,ab. \| 11037 \| Advanced \|  \|  \|  \| \| 3 \| (3D adj3 (print* or model* or manufactur*)).ti,ab. \| 8791 \| Advanced \|  \|  \|  \| \| 4 \| (3-D adj3 (print* or model* or manufactur*)).ti,ab. \| 1720 \| Advanced \|  \|  \|  \| \| 5 \| rapid prototyp*.ti,ab. \| 1344 \| Advanced \|  \|  \|  \| \| 6 \| additive manufactur*.ti,ab. \| 195 \| Advanced \|  \|  \|  \| \| 7 \| stereolithograph*.ti,ab. \| 677 \| Advanced \|  \|  \|  \| \| 8 \| selective laser sinter*.ti,ab. \| 151 \| Advanced \|  \|  \|  \| \| 9 \| selective laser melt*.ti,ab. \| 75 \| Advanced \|  \|  \|  \| \| 10 \| fused deposition model*.ti,ab. \| 89 \| Advanced \|  \|  \|  \| \| 11 \| or/1-7 \| 21668 \| Advanced \|  \|  \|  \| \| 12 \| exp Cardiac Catheterization/ \| 45329 \| Advanced \|  \|  \|  \| \| 13 \| exp Cardiovascular Surgical Procedures/ \| 326759 \| Advanced \|  \|  \|  \| \| 14 \| exp Heart Diseases/ \| 985497 \| Advanced \|  \|  \|  \| \| 15 \| exp Heart Defects, Congenital/ \| 134533 \| Advanced \|  \|  \|  \| \| 16 \| exp Cardiology/ \| 16553 \| Advanced \|  \|  \|  \| \| 17 \| or/12-16 \| 1190088 \| Advanced \|  \|  \|  \| \| 18 \| 11 and 17 \| 635 \| Advanced \|  \|  \|  \| \| 19 \| electrophysiolog*.ti,ab. \| 83231 \| Advanced \|  \|  \|  \| \| 20 \| 18 not 19 \| 576 \| Advanced \|  \|  \|  \| \| 21 \| remove duplicates from 20 \| 555 \| Advanced \|  \|  \|  \| \| 22 \| animals/ not (animals/ and humans/) \| 4243994 \| Advanced \|  \|  \|  \| \| 23 \| 21 not 22 \| 496 \| Advanced \|  \|  \|  \| \| 24 \| limit 23 to yr="2000 -Current" \| 445 \| Advanced \|  \|  \|  \| |
| --- | --- | --- | --- | --- | --- | --- | --- | --- | --- | --- | --- | --- | --- | --- | --- | --- | --- | --- | --- | --- | --- | --- | --- | --- | --- | --- | --- | --- | --- | --- | --- | --- | --- | --- | --- | --- | --- | --- | --- | --- | --- | --- | --- | --- | --- | --- | --- | --- | --- | --- | --- | --- | --- | --- | --- | --- | --- | --- | --- | --- | --- | --- | --- | --- | --- | --- | --- | --- | --- | --- | --- | --- | --- | --- | --- | --- | --- | --- | --- | --- | --- | --- | --- | --- | --- | --- | --- | --- | --- | --- | --- | --- | --- | --- | --- | --- | --- | --- | --- | --- | --- | --- | --- | --- | --- | --- | --- | --- | --- | --- | --- | --- | --- | --- | --- | --- | --- | --- | --- | --- | --- | --- | --- | --- | --- | --- | --- | --- | --- | --- | --- | --- | --- | --- | --- | --- | --- | --- | --- | --- | --- | --- | --- | --- | --- | --- | --- | --- | --- | --- | --- | --- | --- | --- | --- | --- | --- | --- | --- | --- | --- | --- | --- | --- | --- | --- | --- | --- | --- | --- | --- | --- | --- | --- | --- | --- | --- | --- | --- | --- | --- | --- | --- |

| \| **Epub Ahead of Print and In-Process & Other Non-Indexed Citations Ovid MEDLINE(R)**July 18, 2016 \| \| \| \| \| --- \| --- \| --- \| --- \| \| **#** \| **Searches** \| **Results** \| **Type** \| \|  \| \| \| \| \| \| \| \| 1 \| (three dimension* adj3 (print* or model* or manufactur*)).ti,ab. \| 2357 \| Advanced \|  \|  \|  \| \| 2 \| (3D adj3 (print* or model* or manufactur*)).ti,ab. \| 2866 \| Advanced \|  \|  \|  \| \| 3 \| (3-D adj3 (print* or model* or manufactur*)).ti,ab. \| 318 \| Advanced \|  \|  \|  \| \| 4 \| rapid prototyp*.ti,ab. \| 351 \| Advanced \|  \|  \|  \| \| 5 \| additive manufactur*.ti,ab. \| 205 \| Advanced \|  \|  \|  \| \| 6 \| stereolithograph*.ti,ab. \| 194 \| Advanced \|  \|  \|  \| \| 7 \| selective laser sinter*.ti,ab. \| 34 \| Advanced \|  \|  \|  \| \| 8 \| selective laser melt*.ti,ab. \| 46 \| Advanced \|  \|  \|  \| \| 9 \| fused deposition model*.ti,ab. \| 51 \| Advanced \|  \|  \|  \| \| 10 \| or/1-9 \| 5555 \| Advanced \|  \|  \|  \| \| 11 \| cardio*.ti,ab. \| 58522 \| Advanced \|  \|  \|  \| \| 12 \| cardiac*.ti,ab. \| 39669 \| Advanced \|  \|  \|  \| \| 13 \| heart disease*.ti,ab. \| 10680 \| Advanced \|  \|  \|  \| \| 14 \| heart defect*.ti,ab. \| 774 \| Advanced \|  \|  \|  \| \| 15 \| transcatheter*.ti,ab. \| 2369 \| Advanced \|  \|  \|  \| \| 16 \| or/11-15 \| 91525 \| Advanced \|  \|  \|  \| \| 17 \| 10 and 16 \| 164 \| Advanced \|  \|  \|  \| \| 18 \| electrophysiolog*.ti,ab. \| 6362 \| Advanced \|  \|  \|  \| \| 19 \| 17 not 18 \| 156 \| Advanced \|  \|  \|  \| \| 20 \| remove duplicates from 19 \| 156 \| Advanced \|  \|  \|  \| \| 21 \| limit 20 to yr="2000 -Current" \| 151 \| Advanced \|  \|  \|  \| |
| --- | --- | --- | --- | --- | --- | --- | --- | --- | --- | --- | --- | --- | --- | --- | --- | --- | --- | --- | --- | --- | --- | --- | --- | --- | --- | --- | --- | --- | --- | --- | --- | --- | --- | --- | --- | --- | --- | --- | --- | --- | --- | --- | --- | --- | --- | --- | --- | --- | --- | --- | --- | --- | --- | --- | --- | --- | --- | --- | --- | --- | --- | --- | --- | --- | --- | --- | --- | --- | --- | --- | --- | --- | --- | --- | --- | --- | --- | --- | --- | --- | --- | --- | --- | --- | --- | --- | --- | --- | --- | --- | --- | --- | --- | --- | --- | --- | --- | --- | --- | --- | --- | --- | --- | --- | --- | --- | --- | --- | --- | --- | --- | --- | --- | --- | --- | --- | --- | --- | --- | --- | --- | --- | --- | --- | --- | --- | --- | --- | --- | --- | --- | --- | --- | --- | --- | --- | --- | --- | --- | --- | --- | --- | --- | --- | --- | --- | --- | --- | --- | --- | --- | --- | --- | --- | --- | --- | --- | --- | --- | --- | --- | --- |

| **Embase**1974 to 2016 July 18 | | | |  |  |  |
| --- | --- | --- | --- | --- | --- | --- |
| **#** | **Searches** | **Results** | **Type** |  |  |  |
|  | | | | | | |
| 1 | three dimensional printing/ | 1245 | Advanced |  |  |  |
| 2 | (three dimension* adj3 (print* or model* or manufactur*)).ti,ab. | 13944 | Advanced |  |  |  |
| 3 | (3D adj3 (print* or model* or manufactur*)).ti,ab. | 14765 | Advanced |  |  |  |
| 4 | (3-D adj3 (print* or model* or manufactur*)).ti,ab. | 2498 | Advanced |  |  |  |
| 5 | rapid prototyp*.ti,ab. | 1920 | Advanced |  |  |  |
| 6 | additive manufactur*.ti,ab. | 349 | Advanced |  |  |  |
| 7 | stereolithograph*.ti,ab. | 892 | Advanced |  |  |  |
| 8 | selective laser sinter*.ti,ab. | 219 | Advanced |  |  |  |
| 9 | selective laser melt*.ti,ab. | 111 | Advanced |  |  |  |
| 10 | fused deposition model*.ti,ab. | 148 | Advanced |  |  |  |
| 11 | or/1-10 | 31499 | Advanced |  |  |  |
| 12 | heart catheterization/ | 55582 | Advanced |  |  |  |
| 13 | exp cardiovascular surgery/ | 571008 | Advanced |  |  |  |
| 14 | exp heart disease/ | 1528943 | Advanced |  |  |  |
| 15 | exp congenital heart malformation/ | 117364 | Advanced |  |  |  |
| 16 | cardiology/ | 91357 | Advanced |  |  |  |
| 17 | or/12-16 | 1890187 | Advanced |  |  |  |
| 18 | 11 and 17 | 1304 | Advanced |  |  |  |
| 19 | exp electrophysiology/ | 790821 | Advanced |  |  |  |
| 20 | electrophysiolog*.ti,ab. | 110093 | Advanced |  |  |  |
| 21 | 19 or 20 | 824653 | Advanced |  |  |  |
| 22 | 18 not 21 | 1098 | Advanced |  |  |  |
| 23 | remove duplicates from 22 | 1072 | Advanced |  |  |  |
| 24 | (exp animals/ or exp animal experimentation/ or nonhuman/) not ((exp animals/ or exp animal experimentation/ or nonhuman/) and exp human/) | 5953040 | Advanced |  |  |  |
| 25 | 23 not 24 | 956 | Advanced |  |  |  |
| 26 | (conference abstract or conference paper or conference proceeding or conference review).pt. | 3048271 | Advanced |  |  |  |
| 27 | 25 not 26 | 577 | Advanced |  |  |  |
| 28 | limit 27 to yr="2000 -Current" | 529 | Advanced |  |  |  |
| 29 | limit 25 to yr="2015 -Current" | 256 | Advanced |  |  |  |
| 30 | 28 or 29 | 640 | Advanced |  |  |  |
|  | | |  |  |  |  |
